# Contraceptive use among women with no fertility intention in Ethiopia

**DOI:** 10.1371/journal.pone.0234474

**Published:** 2020-06-11

**Authors:** Melash Belachew Asresie, Gedefaw Abeje Fekadu, Gizachew Worku Dagnew

**Affiliations:** Department of Reproductive Health and Population Studies, School of Public Health, College of Medicine and Health Sciences, Bahir Dar University, Bahir Dar, Ethiopia; Ordu University, TURKEY

## Abstract

**Introduction:**

Ethiopia is one of the Sub-Saharan African countries with high unintended pregnancy rate. Every woman in Ethiopia experiences at least one unintended birth. Although there were some studies about contraceptive use among all women in Ethiopia, evidence about contraceptive use among women with no fertility intention was limited. Therefore, this analysis was performed to assess the prevalence of contraceptive use and associated factors among fecund, married reproductive-age women who intended no more children.

**Methods:**

We used the 2016 Ethiopian Demography and Health Survey (EDHS) data collected through a two-stage stratified cluster sampling technique. EDHS was a community based, cross-sectional study conducted from January 18, 2016, to June 27, 2016. A total of 2,859 fecund married reproductive age women with no desire to have more children were included in this study. Both descriptive and logistic regression analysis were performed using STATA V.14. A 95% confidence interval was used to declare statistical significance.

**Results:**

Contraceptive use among fecund married reproductive-age women who want no more children was 51.1% (95%CI: 47.0–55.24%). Visit by health workers at home (AOR = 1.37, 95%CI: 1.02, 1.83), living in Addis Ababa (AOR = 3.38 95%CI: 1.76, 6.37) and having better wealth index (middle (AOR = 1.76, 95%CI: 1.25, 2.47) and being rich (AOR = 1.96, 95%CI: 1.40, 2.74)) were found positively associated with contraceptive use. On the other hand, living in the Somali region (AOR = 0.10, 95%CI: 0.01, 0.85), and being Muslim (AOR = 0.45, 95%CI: 0.30, 0.67) were found negatively associated with contraceptive use.

**Conclusion:**

Contraceptive use among fecund married reproductive-age women with no fertility intention was low compared to their demand. Therefore, to improve contraceptive use, the provision of family planning counseling and information should be strengthened. Further intervention is needed to narrow disparities in contraceptive use among regions and different population groups.

## Introduction

Reducing unintended pregnancy is a public health goal since unintended pregnancy is associated with high population growth, poor maternal and child health [1, 2]. Family planning (FP), is a proven strategy to prevent unintended pregnancies [3–5]. Recent studies identified that contraceptive use could reduce almost 230 million births every year by preventing unintended pregnancy [6, 7]. Another study showed that contraceptives use alone can reduce maternal mortality by 44% [8]. Despite these benefits, contraceptives use among married reproductive-age women remains low. According to the 2017 global report, contraceptive use among these women was 58% globally and in Africa 32% [9]. In developing countries, about 214 million women of reproductive age want to avoid pregnancy but were not using contraceptives in 2018 [10].

A study conducted in 61 countries, identified the age of women, place of residence, wealth status, educational attainment, parity, age at first marriage and fertility preference as the most important factors influencing contraceptive use [11].

The family planning service in Ethiopia was introduced by the Family Guidance Association of Ethiopia in 1966. The FP service at this time was mainly awareness creation due to social and political constraints. Ethiopia developed a national population policy in 1993 to harmonize the rate of population growth and country development and rational utilization of natural resources [12–14].

To accelerate contraceptive and other maternal service use Ethiopia launched the health extension program in 2003. Family planning service is one of the 16 packages in this program [15]. Because of this contraceptive use increased from 8% in 2000 to 36% in 2016. However, 24% of reproductive age women who want to delay or avoid pregnancy were not using a contraceptive [16]. Women’s occupational, awareness about FP, discussion with husband, support from husband, age of women, parity, and wealth status of the household were factors found associated with contraceptive use [17, 18].

The Ethiopian health sector transformation plan targeted to increase the contraceptive prevalence rate to 55% and reduce unmet needs to 10% by the end of 2020 [19]. All fecund and married reproductive-age women who intend to limit their fertility (those who do not want to have more children) are expected to use contraceptives [20]. But the scenario may be different in developing countries [21]. Identifying factors associated with contraceptive among fecund, married reproductive-age women who want no more children is crucial as the incidence of unmet need for contraceptives may be high among this group of women [22, 23]. Although there were studies about contraceptives use and associated factors are available in Ethiopia [24, 25], there are no studies among this specific group of women in Ethiopia. Therefore, this study was done to assess contraceptive use and associated factors among fecund married reproductive-age women who want no more children in Ethiopia.

## Methods

### Data source

We used the 2016 Ethiopian Demography and Health Survey (EDHS) data. The data is a nationally representative, community-based cross-sectional, collected through a two-stage stratified cluster sampling technique from January 18, 2016, to June 27, 2016. The survey was implemented by the Central Statistical Agency at the request of the Federal Minister of Health (HMOH) [16]. The EDHS initially, each region stratified into urban and rural areas yielding 21 sampling strata. Under strata, there were 84,915 enumeration areas. From these, a total of 645 enumeration areas (202 in urban areas and 443 in rural areas) were selected proportionally based on the 2007 Ethiopia population and housing census. Finally, at each enumeration area, 28 households were selected through systematic random sampling technique by using lists get from 2007 EPHC as a sample frame. The survey covered a total of 17,067 households yielding a response rate of 98%.

All reproductive age women (15 to 49 years) who were either permanent residents of the selected households or visitors who stayed in the selected household the night before the survey were eligible for the study. A total of 1, 5683 women aged 15–49 years were interviewed yielding a response rate of 95%. This analysis was restricted to married women with no fertility intention (3,756). Of those, women who were pregnant or amenorrheic (337) and declared in-fecund (560) at the time of the survey were excluded. Finally, 2859 married or in-union fecund married reproductive-age women who want no more children were included in the final analysis.

### Measurements

#### Outcome variable

The 2016 EDHS questionnaire asked all reproductive-age women involved in the survey if they want children in the future to assess their fertility intention. The women responded as they want soon, want later, or want no more. Those women who responded “want no more” were included in this analysis.

The outcome variable for this analysis was contraceptive use, which has two categories (yes or no). Similarly, the 2016 EDHS questionnaire asked all reproductive-age women involved in the survey whether they were using contraceptives at the time of survey including the type of contraceptive. If the woman reported she was using contraceptives (modern or traditional), she was considered as a contraceptive user (yes) and otherwise “no”. Contraceptive type considered as modern methods were male and female sterilization, intrauterine device (IUD), Injectable, oral contraceptive (pills), lactational amenorrhea method (LAM), Standard day’s method (SDM) or a condom). And traditional contraceptive methods were withdrawal and periodic abstain [16].

#### Independent variables

Demographic and socio-economic factors were included in the analysis as independent variables. The demographic variables included were; the age of women (15–34, 35–49), family size (≤4, 5–8, and >9), age of women at first marriage (≤18, 19–24, and ≥25), husband’s fertility desire (both want same, husband wants more, husband wants fewer, don’t know), number of living children (0, 1–2, 3–4, ≥5), number of children ever born (0, 1–2, 3–4, ≥5), knowledgeable on contraceptives (yes, no), and women’s religion (orthodox, Muslim, others). The socio-economic variables included were residence (rural, urban), region, educational status (no, primary, secondary or above), partner’s educational status (no, primary, and secondary or above), working status (not working, working out of a home), partner’s working status (not working, working, don’t know), and wealth index (poor, middle, rich), exposure to FP messages on mass media (no, yes), visit by health workers (no, yes), and visit health facility (no, yes). The exposure to FP messages on mass media was constructed from other data on (if women heard or saw family planning messages from radio, television, magazine, or phone message). The poor wealth index category was created by merging poorer and poorest and the variable rich was constructed by merging richer and richest.

#### Statistical analysis

Weighted data were analyzed using STATA software version 14.1. Frequency distribution and descriptive statistics(proportion, mean±standard deviation(SD)) were calculated. Multicollinearity between independent variables was cheeked before fitting the final regression model. When multicollinearity between two independent variables was detected, one was dropped. Bivariable and multivariable logistic regression analysis were done to identify factors associated with contraceptive use among fecund married reproductive-age women who want no more children. Also, complex survey analysis techniques were employed when computing odds ratios since DHS used a two-stage stratified sampling technique. A 95% confidence interval was used to declare statistical significance. The goodness of fit of the final model was tested by Hosmer-Lemeshow p-value > 0.05.

## Ethical approval

The 2016 EDHS protocol was reviewed and approved by the Federal Democratic Republic of Ethiopia Ministry of Science and Technology and the Institutional Review Board of ICF international (Ethical approval number, Authletter-137912).

## Results

### Socio-demographic and other characteristics of women

A total of 2859 women who want no more children were included in the analysis. The women’s age ranged from 16–49 years with a mean age of 34.7 years (standard deviation(SD) of ± 1 year). About half (53.2%) of the women were aged 35 or more years old and the rest (46.8%) were aged 16–34 years of old. The majority of the women (87.1%) were rural residents. Regarding region, the majority of women (90.0%) were from three regions (Amhara (25.9%), Oromia (42.4%), and South nation and nationality people region (SNNPR) (21.8%)). The rest (10.0%) were from 6 regions (Tigray (4.9%), Somali (0.4%), Beshangul Gumuz (1.0%), Gambella (0.3), Afara (0.3%), and Harari (0.2%)), and two cities (Dire Dawa (0.4%), and Addis Ababa (2.6%)).

Many women (71.0) did not attend formal education. The rest attended primary (23.9% and secondary or more level of education (5.1%). Regarding working status, about 50%) of women and 93% of their husbands were working at the time of the survey. The majority (78.4%) of women reported that they married before their 19^th^ birthday. Most women (74.1%) reported that they did not receive or saw family planning messages through radio, television, magazine, or phone message. Almost half of women (49.0%) reported that they visited a health facility in the 12 months before the survey. About one-third of women (32.9%) reported that they were visited by health workers at home 12 months preceding the survey.

The ideal number of children was four or more for 66.3% of women. Thirteen percent of women didn’t determine the number of children they want to have. About 12% of women want to have 1–3 children. The analysis showed that the mean number of children ever born was 6 (SD ±0.1) which was higher compared to the mean ideal number of children 4.5 (SD ±0.1). A little more than half (58.1%) of women had five or more living children (Table 1).

**Table 1:** Socio-demographic and other characteristics of fecund married women who want no more children in Ethiopia, 2016 EDHS.

Variables	Categories	Frequency n (%)
Residence	Urban	368 (12.9)
	Rural	2,491 (87.1)
Wealth	Poor	1,108 (38.8)
	Middle	607 (21.2)
	Rich	1,144 (40.0)
Working status	Not working	1,440 (50.4)
	Working	1,419 (49.6)
Partners’ educational status	No education	1,418 (49.6)
	Primary	1,100 (38.5)
	Secondary+	318 (11.1)
	Don’t know	23 (0.8)
Religion	Orthodox	1,175 (41.1)
	Muslim	912 (31.9)
	Others*	772 (27.0)
Partners’ working status	Not working	171 (6.0)
	Working	2,654 (92.8)
	Don’t know	34 (1.2)
Family size	≥4	424 (14.8)
	5–8	1,947 (68.1)
	≥9	488 (17.1)
Age at first marriage	≤18	2,240 (78.4)
	19–24	502 (17.6)
	≥25	117 (4.0)
Number of children ever born	0	21(0.7)
	1–2	321 (11.2)
	3–4	669 (23.4)
	≥5	1,848 (64.7)
Number of living children	0	21(0.7)
	1–2	378(13.2)
	3–4	800 (28.0)
	≥5	1,660 (58.1)
Contraceptive knowledge	No	11 (0.4)
	Yes	2,848 (99.6)
Husbands’ fertility desire	Both want the same	1,138 (40.4)
	Husband wants more	753 (26.7)
	Husband wants fewer	230 (8.2)
	Don’t know	696 (24.7)
Visit health facility	No	1457(51.0)
	Yes	1402 (49.0%)

Other* = Protestant, Catholic and traditional

### Contraceptive use

**Fig 1:**
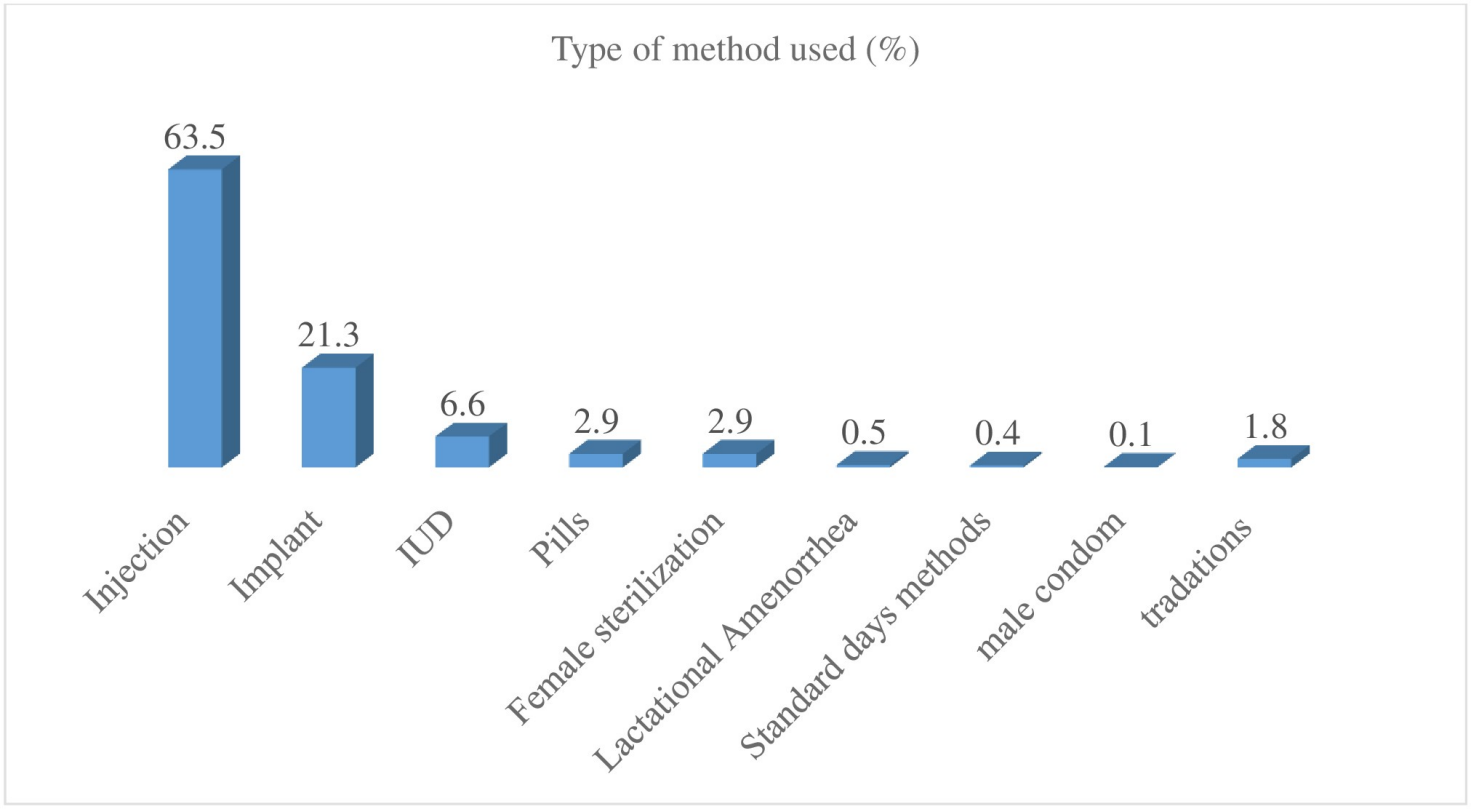
Contraceptive use by methods among fecund married women who want no more children in Ethiopia, 2016 EDHS.

Of all women who want no more children, half of them (51.1%), (95%CI: 47.0, 55.2) were using contraceptives. The majority (50.2%) were using modern contraceptives (Fig 1). However, about 85% wanted to use contraceptives. Most users (69.6%) decided jointly to use contraceptives. About 23% of women decided alone.

### Contraceptive use by women’s characteristics

The proportion of contraceptives was higher among richer women (61.4%) compared to poor women (38.7%) (p<0.001). More women who reported that they were visited by health workers 12 months before the survey (58.4%) were using contraceptives compared to women who were not visited (47.5%) (p = 0.002). The proportion of women using contraceptives (56.1%) was higher among those who reported working at the time of the survey compared to those who were not working (46.2%) (p = 0.002). Contraceptive uptake differed by the number of living children (p = 0.001). About 60% of women who had 1–2 and 3–4 living children were using contraceptives compared to 44.3%, and 45.1% of women who had no living children and those who had five or more children respectively. More urban women (67.7%) were using contraceptives compared to rural women (48.7%) (p<0.001). Women with four or lower family size had significantly higher contraceptive use rate (57.4%) compared to women with nine or more family size (30.5%) (p<0.001). A higher prevalence of contraceptive use was found among women who visited health facilities 12 months prior to the survey (55.2%) compared to women who didn’t visit health facilities (47.2%)(p = 007). A high proportion of women in Addis Ababa (78.0%) were using contraceptives compared to women in Somali region (p<0.001).

There was no statistically significant difference in contraceptive use among fecund married reproductive-age women by age, educational status, age at first marriage, exposure to FP messages on media, and husband’s fertility (Table 2).

**Table 2:** Contraceptive use by women’s characteristics among fecund married women who want no more children in Ethiopia, 2016 EDHS.

Variables	Categories	Contraceptive use n (%)	p-value
Residence	Urban	249 (67.7)	<0.001
	Rural	1213 (48.7)	
Age	15–34	682 (51.0)	0.921
	35–49	779 (51.3)	
Wealth index	poor	429 (38.7)	<0.001
	Middle	330 (54.4)	
	Rich	702 (61.4)	
Educational status	No education	999 (49.3)	0.192
	Primary	379 (55.4)	
	Secondary+	83 (56.6)	
Partner’s education status	No education	628 (44.3)	<0.001
	Primary	634 (57.6)	
	Secondary+	189 (59.4)	
	Don’t know	10 (45.9)	
Religion	Orthodox	693 (59.0)	<0.001
	Muslim	296 (32.5)	
	Others	472 (61.2)	
Partner’s working status	Not working	83 (48.8)	0.039
	working	1371 (51.7)	
	Don’t know	7 (19.0)	
Family size	≥4	243(57.4)	<0.001
	5–8	1069(54.9)	
	≥9	149(30.5)	
Age at 1^st^ marriage	≤18	1156(51.6)	0.823
	19–24	289(49.5)	
	≥25	56(48.3)	
Number of ever born	0	9 (44.8)	<0.001
	1–2	184 (57.4)	
	3–4	419 (62.5)	
	≥5	849 (46.0)	
Number of living children	0	9 (44.3)	<0.001
	1–2	226 (59.9)	
	3–4	477 (59.6)	
	≥5	749 (45.1)	
Husbands’ fertility desire	both want the same	630 (55.3)	0.090
	husband wants more	357 (47.4)	
	husband wants fewer	106 (46.0)	
	Don’t know	326 (46.9)	
Exposed to FP media	No	1054(49.8)	0.164
	Yes	407(54.9)	
Visit health facility	No	687(47.2)	0.007
	Yes	774(55.2)	

### Reason for not using contraceptives

The most common reasons for non-use of contraceptives among fecund married women who intend to have no more children were thinking that contraceptives are fatalistic (22.2%), fear of side effect (14.5%), on breastfeeding (12.2%), menses not returned (9.8%), religious prohibitions, (7.1%), infrequent sex (5.1%), husband and others opposition (2.1%), not having sex (1.9%), lack of access/ too far(1.9%) and available methods inconvenient to use (1.6%).

### Factors associated with contraceptives use

On the bivariable analysis, region, educational status, religion, occupation, wealth index, exposure to FP messages on media, husbands’ fertility desire, visit by health workers, and visiting health facility were associated with contraceptive use among fecund married women who want no more children at p-value 0.2. On multivariable analysis, region, wealth index, religion, and visited by health workers were significantly associated with contraceptive use among fecund married women who want no more children at p-value 0.05. The odds of contraceptive use among women living in Addis Ababa were 3.38 times higher compared to women living in Tigray (AOR = 3.35, 95%CI: 1.76, 6.37). The odds of contraceptive use among women who belonged to the middle and rich wealth index was nearly 2 times higher compared to women who were in the poor wealth index category (Table 3).

**Table 3:** Factors associated with contraceptives use among fecund married women who want no more children in Ethiopia, 2016 EDHS.

Variables	COR (95%CI)	AOR 95%CI
Region		
Tigray	Ref.	Ref.
Afar	0.32(0.14, 0.74)	0.70(0.31, 1.59)
Amhara	1.22(0.78, 1.92)	1.38(0.86, 2.21)
Oromia	0.70(0.43, 1.12)	1.08(0.63, 1.86)
Somalia	0.03(0.01, 0.21)	0.10(.01, 0.85)*
Benshangul Gumuz	0.69(0.40, 1.16)	0.95(0.54, 1.70)
SNNPR	1.42(0.90, 2.26)	1.36(0.72, 2.58)
Gambella	0.91(0.51, 1.64)	1.01(0.50, 2.03)
Harari	0.63(0.36 1.13)	1.94(0.62, 2.30)
Addis Ababa	3.35(2.03, 5.53)	3.38(1.76, 6.37)***
Dire Dawa	0.76(0.42, 1.35)	1.44(0.73, 2.81)
Educational status		
No education	Ref.	Ref. Ref.
Primary	1.28(0.96, 1.70)	1.02(0.75, 1.38)
Secondary+	1.35(0.74, 2.46)	0.70(0.33, 1.51)
Wealth index		
Poor	Ref.	Ref.
Middle	1.89(1.38, 2.59)	1.76(1.25, 2.47)***
Rich	2.52(1.90, 3.33)	1.96(1.40, 2.74)***
Religion		
Orthodox	Ref.	Ref.
Muslim	0.33(0.24, 0.47)	0.45(0.30, 0.67)***
Others	1.10(0.76, 1.590	1.18(0.69, 2.02)
Working status		
Not working	Ref.	Ref.
Working	1.49(1.13, 1.96)	1.30(1.00, 1.69)
Exposed to FP media		
No	Ref.	Ref.
Yes	1.23(0.92,1.64)	0.90(0.65, 1.26)
Visited by a health worker		
No	Ref.	Ref.
Yes	1.55(1.1, 2.06)	1.37(1.02, 1.83)*
Visit health facility		
No	Ref.	Ref.
Yes	1.38(1.09, 1.75	1.21(0.95, 1.55)
Husbands’ fertility desire		
Both want the same	Ref.	Ref.
Husband wants more	0.73(0.52, 1.01)	0.76(0.55, 1.09)
Husband wants fewer	0.67(0.46, 1.02)	0.71(0.47, 1.10)
Don’t know	0.71(0.51, 1.00)	0.83(0.61, 1.14)

Key: Ref. = reference,

* = p-value≤0.05,

** = p-value≤0.01,

*** = p-value≤0.001

## Discussion

Our analysis revealed that the prevalence of contraceptive use among women who want no more children was 51.1%. The majority (63.5%) of women were using injectable followed by implant/Norplant (21.3%), intrauterine device (6.6%), and female sterilization (2.9%). All women are married which means they are more likely to have unprotected sexual intercourse. Therefore, this level of contraceptive use was low because all these women should be protected from unintended pregnancy.

The level of prevalence of contraceptive use in this study was lower than the finding in Indonesia (58.6%) [26] and India (63.4%) [27]. The reason for the low prevalence of contraceptive uptake in Ethiopia might be due to the difference in residence and education. All women who participated in the Indian study were urban dwellers and most of them (69%) were educated. Women's education could help women to understand their rights and responsibilities on the reproductive and sexual issues. Women with a better educational level have better access to health care information from different sources [28–30]. However, the level of prevalence of contraceptive use was higher compared to other studies done in the Oromia region of Ethiopia (16.9%) [31], Nigeria (33.2%) [32], another study in Nigeria(37.6%) [33], and Saudi Arabia 40% [34]. The reason might be traditional beliefs, religious, educational status and cultural differences of study participants [29, 35]. The most frequently mentioned reasons for not using contraceptives were fear of side effects, fatalistic, on breastfeeding, menses not returned, religious prohibitions, infrequent sex, and husband and others opposition. The findings were consistent with other studies done in Ethiopia, Nigeria, and Saud Arabia Arabia [36–39].

The analysis showed that there were disparities in contraceptive use by regions. The proportion of women using contraceptives was higher among women in Addis Ababa (78.0%), in SNNPR (60.1), and Amhara region (56.5%) compared to that in Somali (2.8%) and Afar (25.5%) regions. This difference may due to access to contraceptives and cultural reasons related to fertility. Although the woman wants no more children, her partner or parents in low may prohibit her from using contraceptives. Partner and religious oppositions for contraceptives are more problematic for women in Afar and Somali regions compared to those in Addis Ababa and Amhara region.

More women from urban areas were using contraceptives compared to women from rural areas. This was consistent with other studies [33]. The reason for this difference(low in the rural) might be due to poor access/too far from health facilities. Inadequate access to famil planning service is one of the predominant reasons for the non-use of contraception [40].

Contraceptive uptake was higher among women who reported that they were working outside a home. This finding was consistent with other studies [27, 33]. It could be explained by the fact that women who were working outside the home might be more educated. Women’s education enables them to discuss with their partners and make joint decisions on FP and family size. Also, working women are more likely to receive family planning-related messages in the workplace. Working women are also better empowered compared to those who were not working. Women empowerment is essential to improve maternal health services uptake.

On multivariable analysis, contraceptive use was associated with the region, household wealth index, women’s religion and visited by health workers 12 months prior to the study. Women who were living in Addis Ababa had higher odds of using contraceptives compared to women who were living in Tigray region. This finding was consistent with the findings of previous studies [25, 41–43]. The reason for different among regions might be the educational status of women, most women living in Addis Ababa were educated. Secondly, health service coverage is higher in Addis Ababa compared with Tigray region.

The odds of contraceptive use among women living in Somali region was lower compared to women who were living in Tigray region. The reason for this variation might be due to differences in a partner and religious opposition among women in these regions. In addition, access to health services is lower in Somali region. The religious difference among women in these regions may be the other reason. Negatively relationship between Muslim women and family planning use has been documented by previous studies [29, 44–46]. This study showed that Muslim religion followers were less likely to use contraceptives compared to Orthodox women. This finding was in line with other studies done in Ethiopia [25, 45], Nigeria [33, 44, 46], Ghana [47], and Bangladesh [29]. The reason for this might be the religious opposition for contraceptive uptake may be more problematic for Muslim women compared to the Christian woman [29, 35].

The other finding in this study was that women with a better wealth index had higher odds of using modern contraceptives compared to those with poor wealth index. This finding was in line with studies conducted in Ethiopia [25], Sauda Arabia [34], Nigeria [44, 48], Angola [49], Bangladesh [50], Ghana [47], and Mali [51]. The reason for this might be direct or indirect the costs women may incur to access contraceptives. The other reason might be the biases and misconceptions related to contraceptive use. Women may believe that contraceptives are not favorable for women involved in laborious activities, in which poor women usually engage in. Exposure to family planning messages on mass media exposure may be the other reason, which showed a significant association with contraceptive use in other studies [50, 52–54].

Our study showed that women who were visited by health workers at home were more likely to use contraceptives compared to those who were not visited. This finding was in line with the study done in Ethiopia [25]. The reason might be women received more information about family planning and unplanned pregnancy during the discussion with the health workers at home.

The limitation of this study was that it did not report spousal communication and discussion on FP. Information on spousal communication is an important variable in contraceptive use studies. However, spousal communication information was not included in the EDHS data.

## Conclusions

Contraceptive use among women who want no more children was low compared to their demand. Wealth index, religion, visit by health workers at home, and region were found significant predictors of contraceptive use. Strengthening family planning service accessibility through home visits by health workers with family planning counseling may improve contraceptive use among women. Moreover, impoving the quality of family planning service provided to clients is crucial. The disparity between the regions in contraceptive use should be addressed by strengthing the health systems. More emphasis should be given to poor, and Muslim women at all efforts.
